# Case report: A case of acute postoperative endophthalmitis following penetrating keratoplasty due to carbapenem-resistant *Klebsiella Pneumoniae* and literature review

**DOI:** 10.3389/fmed.2023.1110411

**Published:** 2023-05-17

**Authors:** Ying Yuan, Xiaoyuan Liu, Li Zhou, Wuchun Ding, Liying Zhang, Jinhua Zheng

**Affiliations:** ^1^Department of Ophthalmology, The Affiliated Hospital of Guizhou Medical University, Guiyang, Guizhou Province, China; ^2^School of Clinical Medical, Guizhou Medical University, Guiyang, Guizhou Province, China

**Keywords:** carbapenem-resistant *Klebsiella pneumoniae*, postoperative endophthalmitis, penetrating keratoplasty, corneal donor, keratoconus

## Abstract

A 22-year-old lady underwent penetrating keratoplasty for serious keratoconus. The following day, it was complicated by the development of infectious endophthalmitis. The source of infection was identified as carbapenem-resistant *Klebsiella pneumoniae*. The donor corneal button might be playing a role in infection transmission due to carbapenem-resistant *Klebsiella pneumoniae* in a sputum culture when the donor was still alive. Nosocomial infections were typically severe, rapidly progressive, and difficult to treat. Finally, the patient underwent therapeutic penetrating keratoplasty again with complete resolution of the infection.

## Introduction

Deep anterior lamellar keratoplasty (DALK) has gradually replaced penetrating keratoplasty (PKP) in keratoconus and other stromal disorders. PKP remains the primary treatment for severe corneal diseases. The complications of PKP, such as infection, endophthalmitis, and rejection, were much more frequent than those of DALK. Sources of infection after keratoplasty contain preoperative contaminated donor cornea, postoperative environment, inadequate asepsis during surgery and residual infectious in the recipient (1–3). *Klebsiella pneumoniae* is an opportunistic pathogen during immunocompromised, endotracheal intubation, or antibiotic abuse and is the main reason of nosocomial infections (4). Donor corneas may be contaminated from the hospital environment. Timely diagnosis and the early active and effective infection control are crucial to prevent complications. Herein, we report a case of acute postoperative endophthalmitis after PKP likely due to the donor cornea contaminated with a hospital-acquired organism.

## Case reports

**Figure 1:**
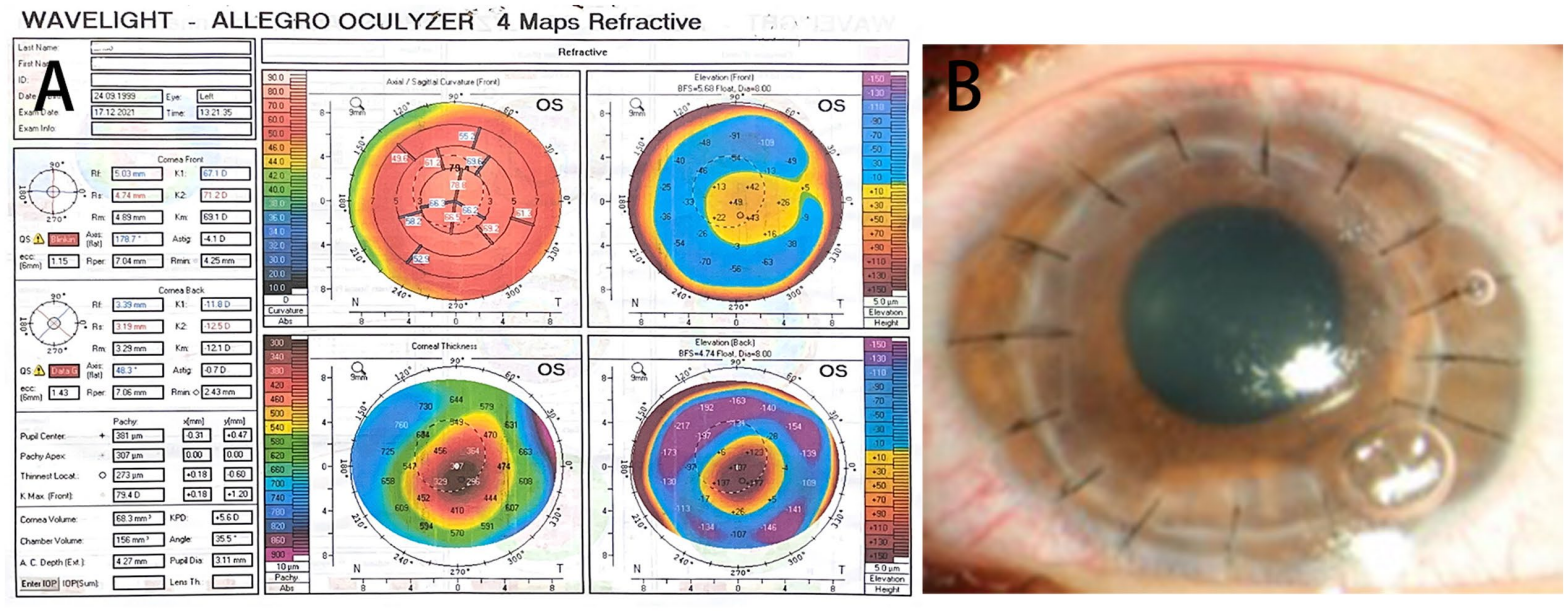
**(A)** The topography showed a paracentral corneal steepening. Sim-K readings are 67.1 D and 71.2 D on the flat and steep axes, respectively, with a corneal thinning point of 273 mm. **(B)** On the first postoperative day (POD), the cornea is transparent and the anterior chamber reaction is mild.

**Figure 2:**
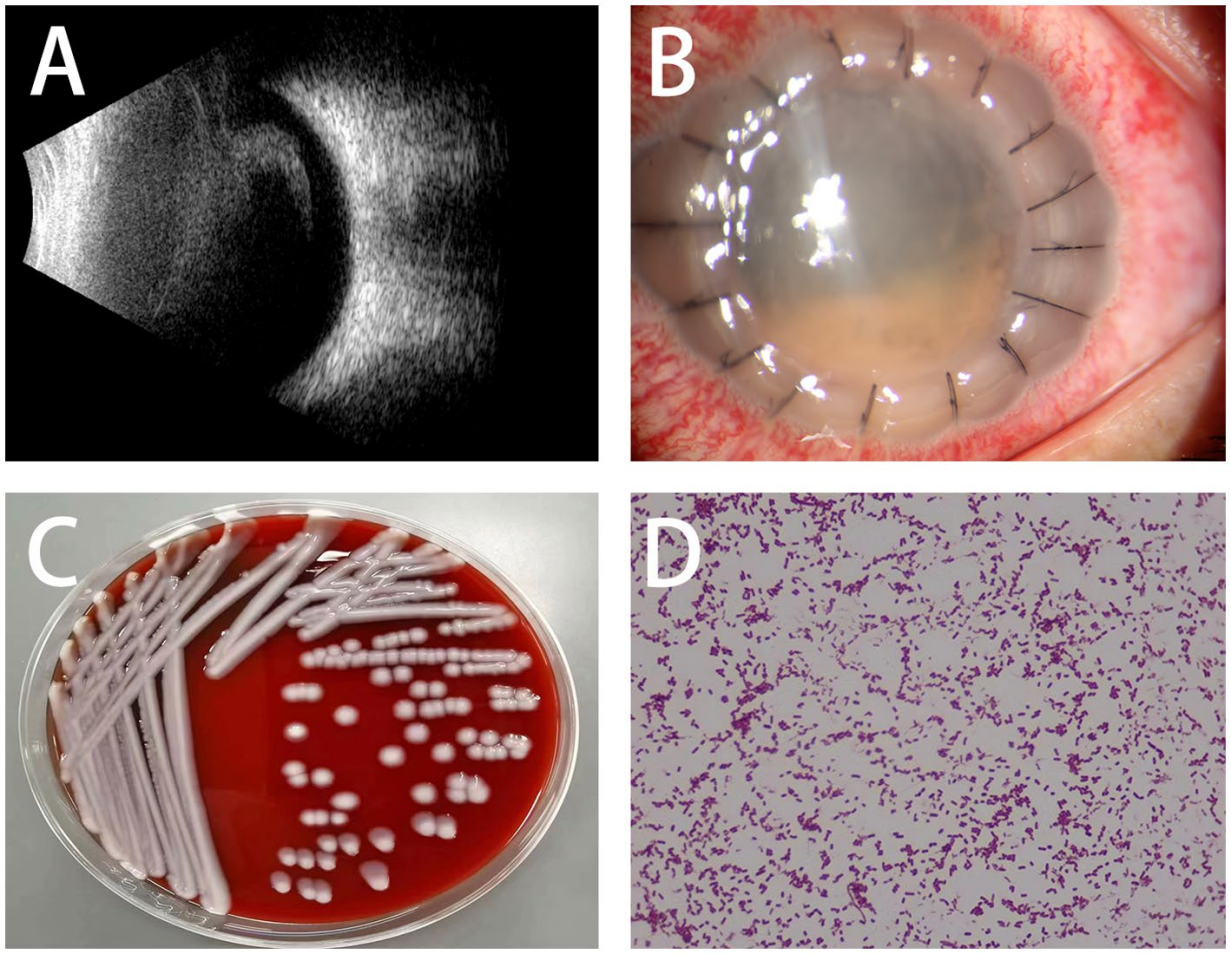
**(A)** Ultrasound shows an increase in echogenicity in the vitreous cavity. **(B)** Clinical picture shows whitish infiltrates in the center of the graft with a small hypopyon. **(C,D)** Bacterial cultures of corneal scrapings and the bacterial smear confirmed the carbapenem-resistant *Klebsiella Pneumoniae* infection.

A 22-year-old lady with severe keratoconus underwent PKP of the left eye. Her best-corrected visual acuity was 20/400, and the preoperative topography showed Amsler–Krumeich stage IV keratoconus in the left eye (Figure 1A). First, DALK was performed, and an 8 mm recipient stromal flap was partially dissected from the surface, but finally PKP had to be converted due to the large perforation of the Descemet membrane. The donor button was cut to 8.25 mm and sutured to the rim of the recipient. The donor cornea was 36 years old and was an *in situ* corneal excision with a transparent cornea in appearance. The surgery had gone quite well, but to our alarm, on the first postoperative day (POD), although the cornea was clear and the anterior chamber reaction was mild, the patient developed a fever with a temperature of 39.5°C (Figure 1B). Routine blood tests showed that the neutrophil was 8.59 × 10^9^ L and the percentage of neutrophils was 87.80%. Systemic vancomycin (50 mg/mL) and ceftazidime (100 mg/mL) were applied immediately via intravenous infusion twice daily considering the possibility of bacterial infection because of increased white blood cells. Furthermore, topical gatifloxacin eye drops increased every hour throughout the day. On the second postoperative day, there was a whitish infiltrate in the center of the graft (Figure 2A), with a little hypopyon and observable vitreous opacity (Figure 2B). The patient’s visual acuity decreased from 20/200 to counting fingers.

**Figure 3:**
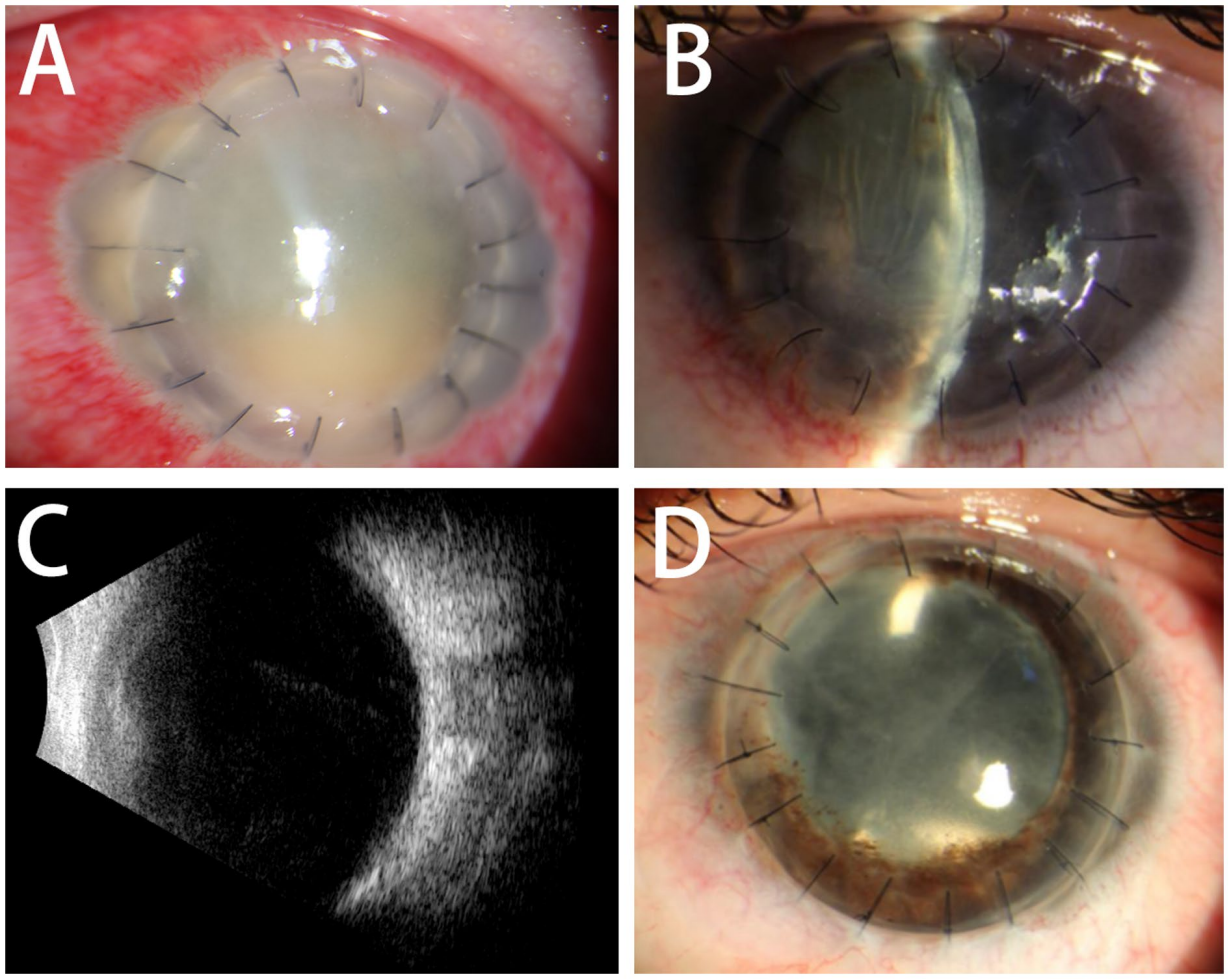
**(A)** The infection was infiltrated to the cornea and anterior chamber of the host. **(B,C)** The day after repeat-PKP, the graft is clear and the anterior segment is quiet with low intraocular pressure and lens opacity. The ultrasound showed vitreous opacity relief. **(D)** Six months later, the aided vision is 20/200 with a pin hole after cataract extraction and intraocular lens implantation.

Bacterial and fungal cultures of corneal scrapings and aqueous humor were tested immediately and which revealed carbapenem-resistant *Klebsiella pneumoniae* (CRKP) (Figures 2C,D), resistant to cefazolin, vancomycin, amikacin, ofloxacin, ciprofloxacin, cefixime, moxifloxacin, chloramphenicol, tetracycline, gentamicin, and cefalexin, but be sensitive to ceftazidime and colistin. Due to the lack of colistin in our hospital, the patient received intraocular antibiotic injections of vancomycin (1.0 mg/0.1 mL) and ceftazidime (2.25 mg/0.1 mL) in the left eye once a day together with topical gatifloxacin, steroids, and atropine once every half an hour. On the third postoperative day, visual acuity was limited to hand motion and whitish infiltrates of the graft worsened with severe edema and anterior chamber exudate on slit-lamp examination, but fever improved with a temperature of 37.5°C and 5.06 × 10^9^ L neutrophils. The treatment regimen was unchanged due to the decrease in temperature and neutrophil count. Five days later, a large diameter PKP (8.75 mm donor and 8.5 mm recipient) was performed due to the extension of the infiltration to the host cornea with anterior chamber opacity (Figure 3A). During surgery, we found that the iris was covered with an exudative membrane, the pupil was fixed, and the anterior lens capsule was opacified. The day after repeat-PKP, the clinical picture improved, with little infiltrate in the graft and exudates from the anterior chamber (Figures 3B,C). The system and intravitreal injections of antibiotics were continued once a day along with topical gatifloxacin, steroids and tacrolimus four times a day, atropine three times a day for 2 weeks. With the advice of the physician in the department of infectious, Meropenem was replaced vancomycin for systemic antibacterial therapy.

Two weeks later, the unaided vision was counting fingers, which improved to 20/1000 with a pin hole. The graft was clear and the anterior segment was quiet, along with low intraocular pressure and lens opacity. The temperature and neutrophil count remained within the normal range. Cataract extraction and intraocular lens implantation were performed 5 months postoperatively (Figure 3D). Six months later, the aided vision was 20/200 with a pin hole. The cornea was transparent without rejection and infection. The image of the retinal was blurred due to the posterior capsule opacity.

## Discussion

*Klebsiella pneumoniae* is a gram-negative bacterium and an important nosocomial pathogen that can cause several infections, especially in hospitalized or immunocompromised individuals, and is associated with high mortality rates, including bloodstream infections, pulmonary infections, liver abscess, ventriculitis, meningitis, ocular infection, and urinary tract infection, which show increasingly frequent acquisition of resistance to antibiotics (5, 6). *Klebsiella pneumoniae* is highly prevalent in East Asian countries, and with an increasing incidence worldwide (7). Carbapenems are the first-line therapy for infections caused by multidrug-resistant *Klebsiella pneumoniae*, particularly extended-spectrum β-lactamase producers. However, carbapenem-resistant Enterobacteriaceae (CRE) strains are challenging because of the resistance to β-lactam agents, and the limitation of the treatment options for CRE-induced diseases (8).

A literature search of databases (PubMed and Google Scholar) was performed using the keyword “Postoperative endophthalmitis and *Klebsiella pneumoniae*,” with a publication date between 2012 and 2022. Of the 1,138 results, 26 case series of endogenous endophthalmitis were reported in 22 articles, only four cases of *Klebsiella pneumoniae*-associated endophthalmitis after cataract surgery, and two cases of *Klebsiella pneumoniae* endophthalmitis after keratoplasty were reported in four articles. Only one case of endogenous endophthalmitis and no case of postoperative endophthalmitis caused by CRKP has been reported (Table 1). Table 1 shows that most cases of *Klebsiella pneumoniae*-associated endogenous endophthalmitis were related to liver abscess, and diabetes mellitus was a major risk factor. Ocular trauma and intraocular surgery are the main causes of exogenous endophthalmitis. Postoperative endophthalmitis after cataract or keratoplasty is common, but vitrectomy is rare.

**Table 1:** Clinical characteristics and treatment of patients with *Klebsiella pneumoniae* endophthalmitis from case reports.

	Author, country, year	Patient sex, age	Underlying diseases	Associated diseases	Right/left eye	Ophthalmic findings	General treatment	Opththalmic treatment	Outcome
Endogenous Endophthalmitis 26 case	Bo Zhao, China, 2021	M, 80	Diabetes mellitus	Liver abscess	Both	VA: NLP, corneal, vitreal haze	iv ^d^ ceftriaxone (2 g/day)	Intravitreal injection with vancomycin and cefoperazone	VA: NLP	
	Pavitra Danapal, Malaysia, 2021	M, 63	Diabetes	Chronic kidney disease	Left	VA: LP, hypopyon, chemosis, vitreal haze	iv ^d^ ceftriaxone, metronidazole	Intravitreal injections (vancomycin and ceftazidime)	VA: NLP
		F, 66	Diabetes	Rectosigmoid carcinoma	Left	VA: HM, corneal, vitreal haze	iv ^d^ ceftriaxone	Intravitreal injections (vancomycin and ceftazidime)	VA: NLP
		F, 42	Diabetes	Bronchial asthma	Left	VA: LP, hypopyon, corneal, Vitreal haze	iv ^d^ ceftriaxone, meropenem	Intravitreal injections (vancomycin and ceftazidime)	VA: NLP
	Jason T. Wells, United States, 2016	F, 67	No	Liver abscess	Left	VA: LP, hypopyon, vitreal haze	iv ^d^ imipenem, vancomycin, and fluconazole	Intravitreal injections (vancomycin and ceftazidime)	VA: NLP
	Jian Lin, China, 2022	M, 51	Diabetes	Liver abscess	Left	VA: NLP, hypopyon, corneal, vitreal haze	iv ^d^ meropenem	Intravitreal injections (vancomycin and ceftazidime)	VA: NLP
	Fadi Hassanin, Saudi Arabia, 2021	F, 55	Diabetes	Renal abscesses	Left	VA: LP, hypopyon, corneal, vitreal haze	iv ^d^ ceftazidime, vancomycin, and metronidazole	Intravitreal injections (vancomycin and ceftazidime)	VA: NLP
								Pars plana vitrectomy	
	Martin Baekby, Denmark, 2018	M, 78	No	Liver abscess	Right	VA: NLP, corneal, vitreal haze	iv ^d^ piperacillin/tazobactam and metronidazole	Intravitreal injections (vancomycin and ceftazidime)	VA: NLP
	Mak CY, China, 2018	M, 67	Diabetes	Colorectal cancer	Left	VA: LP, hypopyon, corneal, vitreal haze	iv ^d^ ciprofloxacin	Intravitreal injections (amikacin and vancomycin)	VA: HM
								Pars plana vitrectomy	
	C. Pichler, Germany, 2016	M, 61	No	Liver abscess	Left	No information	iv ^d^ piperacillin/tazobactam and levofloxacin	No information	No information	
	Chen-Guang Zhang, China, 2022	M, 40	Diabetes	Liver, lung, brain abscess	Left	VA: NLP, hypopyon, corneal, vitreal haze	iv ^d^ meropenem and ceftriaxone	Pars plana vitrectomy	VA: NLP
	Yanquan Liu, China, 2022	F, 60	No	Liver, lung abscess	Left	VA: NLP, hypopyon, corneal, vitreal haze	iv ^d^ imipenem, moxifloxacin, ceftriaxone, azithromycin, meropenem and amikacin	Left eye enucleation	VA: NLP
	Benjamin Kambiz, Ghiam, United States, 2019	M, 34	Diabetes, intravenous drug abuse	Multiple arm and leg abscesses	Right	VA: LP, hypopyon, chemosis, corneal, vitreal haze	iv ^d^ vancomycin and piperacillin/tazobactam	Intravitreal injections (vancomycin and ceftazidime)	VA: LP
								Pars plana vitrectomy	
	Samik Doshi, Canada, 2022	M, 51	Diabetes	Lung and liver abscesses	Right	VA: LP, hypopyon, corneal, vitreal haze	iv ^d^ meropenem and ceftriaxone	Intravitreal injections (ceftazidime)	VA: NLP
	Sung Woong Lim, Republic of Korea, 2020	F, 50	Diabetes	Liver abscess	Left	VA: HM, hypopyon, corneal, vitreal haze	No information	Intravitreal injections (vancomycin and ceftazidime)	VA: 0.08
								Pars plana vitrectomy	
		F, 62	No	Liver abscess	Right	VA: FC, hypopyon, vitreal haze	iv ^d^ ceftriaxone, amikacin, and metronidazole	Intravitreal injections (vancomycin and ceftazidime)	VA: 0.2
								Pars plana vitrectomy	
	Masashi Fujita, Japan, 2019	F, 70		Liver abscess	Right	VA: LP, hypopyon, corneal, vitreal haze	iv ^d^ sulbactam /cefoperazone	Pars plana vitrectomy, ophthalmectomy	VA: NLP
		M, 50	Diabetes	Liver abscess	Right	VA: LP, hypopyon, corneal, vitreal haze	iv ^d^ cefazolin, cefepime	Pars plana vitrectomy	VA: LP
	Ayeshah Abdul-Hamid, United Kingdom, 2013	M, 36	No	Liver abscess	Right	VA: LP, hypopyon, corneal, vitreal haze	Oral ciproflfloxacin twice daily	Intravitreal injections (Amikacin and vancomycin)	VA: NLP
	Darpun D. Sachdev, United States, 2012	F, 58	No	Liver abscess	Right	VA:20/60, hypopyon, corneal, vitreal haze	iv ^d^ levoflfloxacin	Intravitreal injections (ceftazidime)	No information	
	Soumaya Bouhout, Canada, 2021	F, 59	Diabetes	Urinary tract infection	Right	VA: HM, hypopyon, chemosis, corneal, vitreal haze	iv ^d^ meropenem	Intravitreal injections (Ceftazidime and moxifloxacin)	VA: NLP
	Fang Li, China, 2022	F, 69	Diabetes	Liver abscess, purulent meningitis	Right	VA: No information, hypopyon, corneal, vitreal haze	iv ^d^ Meropenem	Intravitreal injections (vancomycin and ceftazidime)	No information
	Harold Henrison Chang Chiu, Philippines, 2018	M, 48	A non-healing wound	Necrotising fasciitis	Left	VA: No information, hypopyon	iv ^d^ piperacillin-tazobactam, vancomycin and ceftazidime	Intravitreal injections (ceftazidime)	VA: LP
								Pars plana vitrectomy	
	Alexander B Crane, United States, 2021	M, 35	Diabetes, cirrhosis and COVID-19	Prostate abscess	Both	VA: LP, Vitreal haze	iv ^d^ ceftriaxone, vancomycin, meropenem and cefepime	Intravitreal injections (vancomycin and ceftazidime)	VA: ODNLP
								Pars plana vitrectomy	OS LP
	Edwin Kamau, United States, 2021	M, 30	Diabetes	Perforated right tympanic membrane, left parotid abscess	Right	VA: LP, hypopyon, corneal, vitreal haze	iv ^d^ ceftriaxone, voriconazole	Intravitreal injections (vancomycin, ceftazidime, and voriconazole)	VA: LP
								Pars plana vitrectomy	
	Seon-Jae Kim, Korea, 2018	F, 55	No	Thyroid abscess, liver abscess	Right	No information	iv ^d^ cefotaxime, metronidazole and amikacin	Intravitreal injections (vancomycin and ceftazidime)	VA: NLP
Multidrug-resistant strains of *Klebsiella pneumoniae* Endogenous Endophthalmitis 1 case	Min Xu, China, 2018	M, 25	Diabetes	Liver abscess	Right	VA: LP, hypopyon, corneal, vitreal haze	iv ^d^ imipenem	Intravitreal imipenem and vancomycin pars plana vitrectomy	VA: FC/40
Carbapenem-resistant strains of *Klebsiella pneumoniae* Endogenous Endophthalmitis 1 case	Yun Zhou, China, 2018	F, 47	Diabetes	Viral pneumonia	Right	VA: LP, hypopyon, chemosis, corneal, vitreal haze	No information	Intravitreal injections (vancomycin and ceftazidime)	VA: NLP
								Pars plana vitrectomy	
Multidrug-resistant *Klebsiella pneumoniae* Postoperative endophthalmitis 6 case	Amit Kumar Deb, Sujatha Sistla, India, 2020	M, 60	No	Following cataract surgeries	Left	VA: HM, hypopyon, corneal, vitreal haze	iv colistin	Intravitreal injections (vancomycin, ceftazidime and colistin)	VA: 20/20
								Pars plana vitrectomy	
	Shekhar Sanghi, India, 2014	No information	1. Phthisis bulbi	Following cataract surgeries	Left	VA: 20/50, hypopyon, corneal, vitreal haze	No information	Intravitreal injections (vancomycin, amikacin, imipenem)	VA: LP
			2. No		Left	VA: LP, hypopyon, corneal, vitreal haze			VA: LP
	3 case		3. Phthisis bulbi		Right	VA: LP, hypopyon, corneal, vitreal haze		pars plana vitrectomy	VA: NLP
	Mukesh Taneja, India, 2016	M, 67	No	Following Descemet-stripping endothelial keratoplasty	Left	VA: LP, hypopyon, corneal, vitreal haze	iv colistin	Intravitreal injections (vancomycin, ceftazidime and colistin)	VA: 20/252
	Bajracharya Leena, Nepal, 2015	F, 47	No	Following deep anterior lamellar keratoplasty	Left	VA: HM, hypopyon, corneal haze	iv ^d^ imipenem	Therapeutic penetrating keratoplasty	VA:6/18

Infectious endophthalmitis is one of the most devastating complications of intraocular surgery. There are few reports of endophthalmitis after lamellar keratoplasty and endothelial keratoplasty, most of which are related to fungal infections. The bacterial species that cause endophthalmitis are an overwhelming preponderance of Gram-positive organisms (approximately 94%), with only 6% of Gram-negative bacteria from a large set of microbiological data (9). Approximately 5.2% of multidrug-resistant strains of *Klebsiella pneumoniae* have been isolated from patients with endophthalmitis. *Klebsiella pneumoniae*-associated endophthalmitis is a minority, however, it generally has worse visual acuity outcomes with high enucleation rates, despite treated with appropriate antibiotics (10). *Klebsiella pneumoniae* also has been shown to induce a significant proinflammatory response in the retinal pigmented epithelium and cause irreversible damage to retinal photoreceptor cells in 12 h at the earliest, probably contributing to the rapid progression and poor prognosis of associated endophthalmitis (11). Multidrug-resistant Klebsiella strains, therefore, result in a greater challenge in management and often confer non-susceptibility to at least one agent in three or more categories of antimicrobials that require treatment with uncommon antibiotics (12, 13).

Our case was donor-related endophthalmitis in which the same organism was isolated from the patient (cornea or anterior chamber) and the donor cornea (cornea preservation fluid/corneoscleral rim preserved after keratoplasty). The donor was a male patient with severe cerebral hemorrhage who had a positive sputum culture for CRKP during the 20 days of coma, with a negative tracheal cannula sputum culture again after antibiotic treatment. The cornea and aqueous humor of the donor were transparent when the cornea was removed. No positive results were found in the culture of corneal preservation solution after the cornea was donated and the transplant surgery was performed strictly according to the requirements of the aseptic operation the next day. However, there was a serious infection on the second postoperative day. Despite timely antibiotic treatment, the patient’s prognosis was poor. A vitrectomy could not be performed due to an opaque cornea. Finally, the patient underwent repeat PKP and cataract extraction to control infection and improve vision. Fortunately, the patient did not develop any systemic infections other than eye infections. It is worth mentioning that the donor’s cornea was transplanted to another patient on the same day without infection.

Although the use of antibiotics in storage media has reduced the donor contamination rate substantially, donor contamination still poses a risk of endophthalmitis and graft infections in the recipients. The positive donor rim culture substantially increases the risk of endophthalmitis, and the risk of endophthalmitis also increases with increasing preservation time of the cornea and the harvesting of the graft from donors with systemic infectious disease (14). In order to avoid the occurrence of the infection, we think the timely and rapidly culture of donor corneal storage medium before transplantation is still necessary. Moreover, it is necessary to avoid PKP or corneal endothelial transplantation when selecting corneal donors with nosocomial infections, particularly in the absence of sensitive antibiotics.

In conclusion, endophthalmitis after PKP is a rare complication whose outcome depends on aggressive and precocious treatment. Identification of early signs of infection is mandatory in patients undergoing PKP to prevent further global involvement.

## Data availability statement

The original contributions presented in the study are included in the article/supplementary material, further inquiries can be directed to the corresponding authors.

## Ethics statement

Written informed consent was obtained from the individual(s) for the publication of any potentially identifiable images or data included in this article.

## Author contributions

JZ and LyZ designed this study. YY and XL performed the literature search, and wrote the manuscript. LiZ and WD performed clinical examination and clinical data collection. All authors contributed to the article and approved the submitted version.

## Funding

This study was supported by the National Natural Science Foundation of China [Grant Numbers: 82060173] and Science and Technology Support Program of Guizhou Province [No. Qian Ke He Support Plan 20204Y145]. Science and Technology Plan of Guiyang [Zhu Ke contract 2019 9-1-21].

## Conflict of interest

The authors declare that the research was conducted in the absence of any commercial or financial relationships that could be construed as a potential conflict of interest.

## Publisher’s note

All claims expressed in this article are solely those of the authors and do not necessarily represent those of their affiliated organizations, or those of the publisher, the editors and the reviewers. Any product that may be evaluated in this article, or claim that may be made by its manufacturer, is not guaranteed or endorsed by the publisher.
